# Laparoscopic cholecystectomy: review of 1430 cases in Cure International Hospital, Kabul, Afghanistan

**DOI:** 10.1186/s12893-021-01342-9

**Published:** 2021-09-17

**Authors:** Wais Farda, Mohammad Kamal Tani, Richard G. Manning, Mohammad Samim Fahmi, Nasratullah Barai

**Affiliations:** 1 General Surgery Department, Isteqlal Hospital, Alaudin Square, Darulaman Road, Kabul, Afghanistan; 2 CURE International Hospital, Darulaman Road, Kabul, Afghanistan; 3 Be Team International Harrisburg, Harrisburg, PA USA

**Keywords:** Laparoscopic, Cholecystectomy, Afghanistan

## Abstract

**Background:**

Laparoscopic cholecystectomy (LC) is the gold standard for the treatment of cholelithiasis in most countries of the world. The objective of this study was to evaluate the outcomes of LC in the surgery department of Cure International Hospital, Kabul, Afghanistan.

**Methods:**

A retrospective study was conducted on 1430 LC cases performed by the general surgery department of Cure International Hospital. Data was collected from patient files and the operation theatre registry for whom LC was performed during January 2008 through December, 2019.

**Results:**

Mean age was 45.77 ± 13.45 years (14–90 years), with male/female ratio of 1:4.7. One third (33%) had comorbidities. Most of patients (~ 97%) were classified as ASA grade I and II. Of all patients, 26.8% of males and 13.2% of females had gallbladder inflammation (OR = 2.203, 95% CI 1.56–2.61, P = 0.000). Overall mean duration of anesthesia was 75 ± 25.6 min. The conversion rate to OC was 4.6% (N = 66), most commonly dense adhesions at Callot’s triangle (3.8%). The intraoperative complication rate was 17.5% (N = 249), where bile/stone spillage was the most common indication (N = 235, 16.4%). Immediate postoperative complication rate was 2.4% (N = 35). Average length of stay (ALOS) after LC was 2.23 ± 1.43 days (1–19 days).

**Conclusion:**

This study shows that elective LC can be performed safely in Afghanistan with comparable outcomes in terms of complications, conversion rates, and ALOS to other countries of the region and the world. Proper case selection and careful preoperative evaluation and management can decrease further conversion, intra- and postoperative complications.

## Background

Since the introduction of laparoscopic cholecystectomy (LC) by Prof Dr. Erich Mühe in 1985 in Germany, followed by Phillipe Mouret in 1987 and Francois Dubois in 1988 in France, LC rapidly gained popularity and is now the procedure of choice in the surgical management of symptomatic gall bladder disease. In early trials, LC was recommended only for elective cholecystectomy in uncomplicated cases [1–5].

LC has numerous advantages and can be performed as a day case surgery. LC-related complications include bleeding from the gallbladder bed or the cystic artery and biliary complications, i.e., spilled gallstones, biliary leak, and common bile duct injury [6–10]. Conversion rate to open cholecystectomy is variable and has been reported from 1.9 to 14.7%, and intraoperative complication rate was 8.3–43% in various series [2–17].

It is noteworthy that the data regarding the safety of daycare LC came from developed countries with well-established norms for daycare surgery. It should not be extrapolated to surgical practice everywhere [8]. However, several studies show that LC can be successfully introduced in developing and low-income countries with satisfactory results [3, 6–10, 15–17].

Mehraj et al. suggested that proper training, case selection, technique, and visual equipment were the key factors to ensure good results [7]. In a comparative study, Brekalo et al. showed that it was possible to achieve satisfactory results in a poorly developed country with little resources and that the human factor played a major role [3]. Straub et al. demonstrated in Mongolia that teaching new and complex surgical skills in a short but dedicated time frame was feasible and rural surgeons could be trained successfully to perform LC safely [17].

However, in some studies, initial capital investment requirements, a longer learning curve, lack of resources, education, supply chain of laparoscopic equipment, technical support, and supportive services, notably endoscopic retrograde cholangiopancreatography and percutaneous transhepatic cholangiography, are shown as the main constraints of performing LC in resource-limited countries [9, 16, 18]. To overcome financial constraints and to make LC affordable to rural population, Gnanaraj et al. have developed a relatively inexpensive, gasless laparoscopy technique using modified reusable open surgical instruments through flexible gel ports under spinal anesthesia [19].

Because they believe that laparoscopic surgery in low- and middle-income countries offer the same advantages as in high-income countries, Alfa-Wali et al. have suggested that a shift towards LC and other new surgical techniques should be encouraged and promoted [20]. In a systematic review, Chao et al. found that laparoscopic procedures could be affordable and patient costs similar to laparotomy in developing countries while decreasing hospitalization and risk of infection [6]. In a 102 LC series performed for the first time in Cure International Hospital in Kabul, Afghanistan, Manning, and Aziz reported overall morbidity and mortality rates compared with rates reported in the developed and developing countries. Their technical complication rate after LC was 3.9% and higher than what was reported elsewhere. Management of these complications required laparotomy due to a lack of supporting radiology and endoscopy services [16]. However, questions were raised regarding the affordability of initial capital expenditure, maintenance of the equipment, reliability of the supply chain, lack of running water, a steady supply of oxygen and electrical power, and a shortage of certified surgeons and anesthesiologists in district and provincial hospitals [21].

LC was introduced more than three decades ago in Europe and the US and revolutionized hepatobiliary surgery, but it didn’t come to Afghanistan until 2006. While LC is the gold standard for cholelithiasis in most countries globally, it is less common in Afghanistan due to limited resources.

This study compares outcomes (complications, length of anesthesia, and length of stay) of LC in Afghanistan to show its safety compared to similarly reported outcomes in other developing and developed countries. It provides evidence for Afghanistan’s surgical society to develop LC as an alternative to open cholecystectomy in its’ specialty hospitals, to develop LC-related training programs for surgeons, biomedical technicians, anesthetists, and nurses, and to invest in laparoscopic infrastructure.

## Methods

### Study design

A retrospective observational study was conducted on laparoscopic cholecystectomy (LC) cases performed at the Cure International Hospital, Kabul, Afghanistan. Data was collected from patient files and the operation theatre registry using a data extraction form designed for the study.

### Study population and sample

The study population was all patients (N = 1430) for whom laparoscopic cholecystectomy has been performed in Cure International Hospital from January 2008 through December 2019.

### Variables and data collection

The data was collected retrospectively from the patient files and anesthesia registry books and entered into a database. Demographic information, surgery, anesthesia, and outcome-related measures were analyzed.

### Data analysis

The data was analyzed using IBM SPSS 25. Descriptive statistics including frequencies and percentages and statistical tests including Chi-square test and ANOVA were used to assess associations.

### Preoperative criteria for patient selection

Patients with no previous upper abdominal surgery, bilirubin < 1 mg/dl and a clear CBD (no stone/obstruction or any lesion in CBD in ultrasonography report) and those without severe chronic respiratory problems were selected for laparoscopic cholecystectomy.

### Preoperative workup

- History and physical examination.
- Abdomen-pelvic ultrasonography.
- CBC, urine routine examination, viral tests (Hepatitis B, Hepatitis C, HIV), LFT (bilirubin, ALT, AST), Serum creatinine.
- Patients > 40 years or patients with comorbidities: all of the above plus ECG and Chest X-ray.
- Diabetics: all of the above plus FBS and Hb1Ac.

### Surgical technique

For LC Stroz laparoscopy set (monitor, camera and video system with 30° 10 mm scope, automatic CO_2_ insufflator, light source), electrosurgical unit (unipolar/bipolar), suction machine, with reusable trocars (10 mm and 5 mm, Hasson trocars; sterilized in Cidex^®^ solution) and basic laparoscopy instruments set was used. LC was done in general surgery operating room allocated for laparoscopic surgery. LC was performed by general surgeons trained by foreign surgeons and those who were trained afterwards in Cure International Hospital, Kabul. A consultant anesthesiologist visited all patients and assigned the ASA grade for them. General anesthesia with endotracheal intubation was given to the patient. Patients were positioned on the table in reverse Trendelenburg with left lateral tilt. LC was done using standard four ports or three port technique. At the start, 4 port technique was used for all LC patients, but in the last 3 years, 4 port technique was used when there were severe adhesions in the subhepatic area or a hanging gall bladder obscuring the Callot’s triangle view. Pneumoperitoneum was created by open Hasson’s technique and maintained at an intra-abdominal pressure of 10–14 mmHg. A 10 mm supra umbilical camera port, 10 mm epigastric port, and two 5 mm ports were used, one each in the midline between the supra-umbilical and epigastric ports, and the other port in the anterior axillary line. A three port (without the axillary port) was also used infrequently. Metallic clips were used to secure the cystic duct and artery. Diathermy was used for dissection as well as hemostasis. The gallbladder was removed through the supra-umbilical port. The supra-umbilical port was closed in two layers, and all other wounds were closed in a single layer.

## Results

The mean age of LC patients was 45.77 ± 13.5 years, and the mean age of men was found 5 years greater than that of women. The ratio of males to females was 1:4.7. One third (33%) of the patients had comorbidities. The most common comorbidities were hypertension, diabetes mellitus, pulmonary and cardiac diseases. 20% of patients with comorbidities had more than one comorbidity. Comorbidity rates were similar for gender, (37% of males and 32% of females) and across all age groups (P = 0.141). Gallbladder stone was reported in the abdominal ultrasonography of all patients, while gallbladder inflammation was reported in only 15% of patients. Gallbladder inflammation was more common in males, 26.8% than females, 13.2%, (OR = 2.203, 95% CI 1.56–2.61, P = 0.000). WBC count at the time of admission was categorized as equal or below and above10,000/mm^3^, and similar at the time of admission for both genders (P = 0.942). The majority of patients (~ 97%) were classified as ASA I and II. Only 52 patients (3.6%) were classified as ASA III. Demographic and clinical statistics are presented in Table 1.

**Table 1:** Demographic and Clinical Statistics

Variables	Male 250 (17.5%)		Female 1180 (82.5%)		Total 1430	
	N	(%)	N	(%)	N	(%)
Age						
15–30	22	(8.8%)	207	(17.5%)	229	(16.0%)
31–50	114	(45.6%)	582	(49.3%)	696	(48.7%)
51–70	106	(42.4%)	372	(31.5%)	478	(33.4%)
71 and over	8	(3.2%)	19	(1.6%)	27	(1.9%)
Mean ± SD	49.5 ± 13.1		44.98 ± 13.4		13.75 ± 13.5	
ASA grade						
I	124	(49.6%)	647	(54.8%)	771	(53.9%)
II	106	(42.4%)	501	(42.5%)	607	(42.4%)
III	20	(8.0%)	32	(2.7%)	52	(3.6%)
Prior abdominal surgery	14	(5.6%)	86	(7.3%)	100	(7.0%)
Comorbidities^a^	93	(37.2%)	382	(32.4%)	475	(33.2%)
Sonogram finding^b^						
GB stone	250	(100%)	1180	(100%)	1430	(100%)
GB inflammation	67	(26.8%)	156	(13.2%)	223	(15.6%)
GB mass	1	(0.4%)	0	(0.0%)	1	(0.1%)
Admission WBC						
≤ 10,000/mm^3^	210	(84.0%)	989	(83.8%)	1199	(83.8%)
> 10,000/mm^3^	40	(16.0%)	191	(16.2%)	231	(16.2%)

^a^Some patients have more than one comorbidity

^b^Some patients have more than one sonogram finding

For most of the LC procedures (~ 88%), afour incision/port configuration was used, and its pattern was similar for both sexes (P = 0.823). Almost all patients (99.9%) had gallbladder stones found during the operation. The only patient without a gallbladder stone had a gallbladder polyp instead. Five patients had a gallbladder polyp with stones. Gallbladder inflammation was found in 198 cases (124 acute and 74 chronic). Three cases with CBD stones, one of which had Mirrizi syndrome, were diagnosed during LC. Their preoperative sonogram and LFT were unremarkable. All these cases were converted to OC. The patient with Mirrizi syndrome had a fistulation between Harmann’s pouch and CBD, which was managed with cholecystectomy, CBD exploration and T-tube insertion. For the other two cases, CBD stones were removed by CBD exploration and insertion of a T-tube. There was only one patient with GB mass, which was sent for histopathology study, and the biopsy report was differentiated adenocarcinoma of the gall bladder. The overall mean duration of anesthesia was 75 min (SD = 25.6) and slightly longer in males than females (P = 0.000). The mean duration of anesthesia in LC cases without conversion to open was 73.3 ± 23.3 min, while it was much longer in LC cases that were converted to open cholecystectomy (110.98 ± 41.0 min) (P = 0.000). However, the duration of anesthesia was similar in LC cases with (77.48 ± 30.3) or without (74.53 ± 24.3 min) intraoperative complications (P = 0.098). The duration of anesthesia was longer in LC cases with post-operative complications (89.71 ± 36.2 min) than those without post-operative complications (74.67 ± 25.2 min), which was statistically significant (P = 0.001).

An abdominal tube drain was placed for 59 (4.1%) patients, most commonly for cases with intra-operative complications (N = 39, 15.6%) compared to uncomplicated cases (N = 20, 1.7%) (P = 0.000). A drain was placed in 22 males (8.8% of male patients) and 37 females (3.1% of female patients), which is statistically significant (P = 0.000). The overall conversion rate to OC was 4.6% (N = 66), with a similar pattern in both male and female patients (OR = 0.647, 95% CI 0.36–1.12; P = 0.139), ASA grades (P = 0.301), WBC count at admission (OR = 1.162, 95% CI 0.61–2.21; P = 0.647) and comorbidity (OR = 1.069, 95% CI 0.63–1.82; P = 0.805). Surgery-related statistics are presented in Table 2.

**Table 2:** Surgery related statistics

Variables	Male 250 (17.5%)		Female 1180 (82.5%)		Total 1430		P value
	N	(%)	N	(%)	N	(%)	
Incision/port							
3 ports	32	(12.8%)	207	(12.3%)	177	(12.4%)	0.823
4 ports	218	(87.2%)	582	(87.7%)	1253	(87.6%)	
Mean duration of anesthesia (min) ± SD	80.7 ± 29.0		73.9 ± 24.7		75.04 ± 25.6		0.000
Drain placed	22	(8.8%)	37	(3.1%)	59	(4.1%)	0.000
Converted to OC	16	(6.4%)	50	(4.2%)	66	(4.6%)	0.139
Intraoperative complications	55	(22.4%)	194	(16.4%)	249	(17.4%)	0.035
Postoperative complications	6	(2.4%)	29	(2.5%)	35	(2.4%)	0.957
ALOS (days) ± SD	2.25 ± 1.4		2.22 ± 1.4		2.23 ± 1.4		0.814

The conversion rate was 26.3% among patients with gallbladder inflammation (29.8% for acute and 20.3% for chronic cholecystitis). While conversion rate was significantly higher among patients preoperatively diagnosed with gallbladder inflammation by ultrasonography (OR = 11.44, 95% CI 6.76–19.34; P = 0.000), it was much higher for acute cholecystitis (OR = 18.727, 95% CI 10.997–31.893; P = 0.000) than for chronic cholecystitis (OR = 6.505, 95% CI 3.458–12.240; P = 0.000). Of patients with prior abdominal surgery (N = 100), none was converted to OC. Indications for conversion are listed in Table 3.

**Table 3:** Indications for conversion to OC

Indications for conversion	N	(%)
Dens adhesions at Callot’s triangle	54	(3.8%)
Bleeding-vascular/GB bed	5	(0.4%)
CBD stone/obstruction	1	(0.1%)
CBD stone + Mirrizi syndrome	1	(0.1%)
CBD stone + bleeding	1	(0.1%)
CBD injury	3	(0.2%)
Duodenal injury	1	(0.1%)
Total	66	4.6%

Intraoperative complications occurred in 249 patients (17.4%), where bile/stone spillage was the most common intra-operative complication. Intraoperative complications are listed in Table 4. There were ten complications (0.7%) requiring conversion to OC. While bile/stone spillage in this series was higher than what is reported in other studies, bleeding and organ injury in this series was lower or comparable to other studies. All the bile spilled during the operation was managed by suction and irrigation with NS. The intraoperative complication rate was significantly higher in male than in female patients (OR = 1.47, 95% CI 1.05–2.05, P = 0.024). The intraoperative complication rate was not significantly different in terms of the number of incisions/port (P = 0.200), mean duration of anesthesia (P = 0.98), and mean of hospital stay after the operation (P = 0.326). However, the intraoperative complication rate was significantly higher among cases with chronic cholecystitis (OR = 1.19, 95% CI 1.02 – 1.38, P = 0.004), those with prior abdominal surgery (OR = 1.14, 95% CI 1.01 – 1.29, P = 0.009), and age older than 70 (N = 11, 40.7%, P = 0.001). There were 1153 symptomatic patients, from whom 249 (21.6%) suffered intraoperative complications. There was no intraoperative complication in asymptomatic cases. Only Immediate postoperative complications were reported because a significant number of patients came from other provinces and did not return for follow-up or contacted the hospital by phone to inform about any complications. The overall immediate postoperative complication rate was 2.4% (N = 35), where the most common complication was bleeding that required transfusion or reoperation. Some patients had more than one postoperative complication, where in most cases, bleeding was one of the complications. Postoperative complications based on Dindo et al. classification [22] is shown in Table 5.

**Table 4:** Intraoperative complications

Complications during surgery	N	(%)
Bile/stone spilled	235	(16.4%)
Port-site bleeding requiring suture	4	(0.3%)
Hepatic fossa bleeding requiring conversion	4	(0.3%)
Other bleeding requiring conversion	2	(0.1%)
CBD injury	3	(0.2%)
Duodenal injury	1	(0.1%)
Total	249	17.4%

**Table 5:** Postoperative complications

Postoperative complications grade (Dindo et al.)	N	(%)
I	3	(0.2%)
II^a^	25	(1.7%)
IIIa^a^	10	(0.7%)
IIIb^a^	10	(0.7%)
Total	48	(3.3%)

^a^Some patients had more than one postoperative complication

From the 12 cases with bile leakage (0.9%) that were diagnosed postoperatively, one was managed by laparoscopic drainage of bile collection and drain placement, three of them were managed by percutaneous drainage and drain placement under local anesthesia, and the remaining eight cases (0.6%) were managed without any intervention. Postoperative complications were similar in terms of sex (OR = 0.98, 95% CI 0.40–2.38, P = 1.000), presence of comorbidity (OR = 1.12, 95% CI 0.60–2.39, P = 0.717), prior abdominal surgery (P = 0.749), number of incision/ports (P = 0.171), intraoperative complications (OR = 0.98, 95% CI 0.40–2.38, P = 0.586) and conversion to OC (OR = 0.51, 95% CI 0.15–1.69, P = 0.217). Postoperative complications were significantly higher in ASA grade III than ASA grade I and II (P = 0.43), and WBC count > 10,000/mm^3^ at admission (P = 0.002).

ALOS, after all cases, were 2.23 ± 1.43 days (1–19 days). ALOS was 2.15 ± 1.3 days for LC cases not converted to OC and 3.67 ± 2.7 days for cases that were converted to OC. About a third of patients (N = 426) were discharged after 1 day, while more than half of them (N = 826, 57.8%) stayed for 2–3 days; only 178 patients (12.4%) stayed more than 3 days in the hospital after LC. ALOS after LC was similar for both sexes and those with intraoperative complications (P = 0.326); but it was significantly higher in patients who were classified as ASA grade III before LC (P = 0.001), had comorbidities (P = 0.001), four incision/port approach (P = 0.000), had drains placed after LC (P = 0.000), LC converted to OC (P = 0.000), who suffered postoperative complications (P = 0.000). Fortunately, there was no mortality in this series.

## Discussion

Since the introduction of laparoscopic cholecystectomy (LC), it rapidly gained popularity, almost replacing open cholecystectomy in many countries [1–5]. Several studies showed that LC can be successfully introduced in developing and low-income countries, with satisfactory results [3, 6–10, 15–17]. However, in Afghanistan, despite the fact that LC was introduced more than a decade ago, most cholecystectomies are done through an open approach due to limited equipment, support services and the manpower required for LC [16, 21].

In our series. The mean age was 45.8 ± 13.5 years, where most of the patients were in 31–50- and 51–70-year age groups. Gender and age distribution in our study population were similar to other studies published on LC elsewhere [3, 6–10, 15–17].

**Fig. 1:**
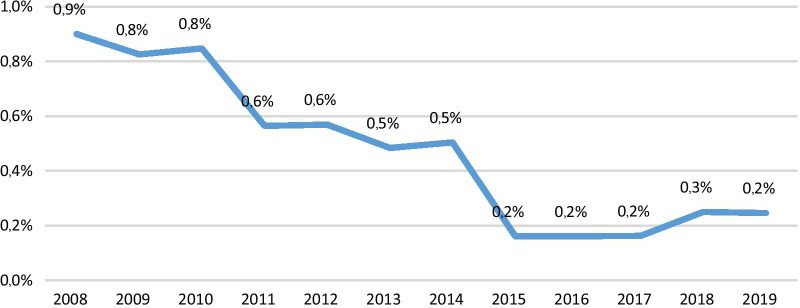
Conversion rate trend

Overall conversion rate to OC in this study was 4.6%, with a similar pattern for both sexes, ASA grades, WBC count at admission and comorbidity, and was comparable with conversion rates reported in various studies (1.9–8.09%) [2–4, 7–10, 15–17]. However, the conversion trend in our study showed a decreasing rate over the years (Fig. 1). While the conversion rate was significantly higher among patients preoperatively diagnosed with gallbladder inflammation by ultrasonography (OR = 11.44), it was much higher for acute cholecystitis (OR = 18.727) than for chronic cholecystitis (OR = 6.505); this can help in selection of cases for LC and predicting the conversion in such patients.

The most common cause of conversion in this study was dense adhesions at Callot’s triangle (81.8%). Other studies [7, 11, 12, 15] also reported adhesions of the Callot’s triangle as the most common cause of conversion.

The intraoperative complication rate in this study was 17.4% (N = 249), where bile/stone spillage was the most common intra-operative complication. The intraoperative complication rate was significantly higher in male than in female patients, but similar in terms of the number of incisions/ports, mean duration of anesthesia, and mean of hospital stay after the operation. Intraoperative complications have been reported in various series from 8.3 to 43%, where the most common intraoperative complications were reported as iatrogenic perforation of gallbladder, bleeding and spilled gallstones [11–14]. While bile/stone spillage in this series was higher than what is reported in other studies, bleeding and organ injury in this series was lower or comparable to other studies. CBD and bowel injury are important intraoperative complications. CBD injury rate has been reported from nil to 1.5% of LCs in various series [3, 4, 10–14, 17]. CBD and bowel (duodenal) injury rate in this study was 0.2% and 0.1% respectively, which is comparable with other studies [10, 12, 14, 16].

Only Immediate postoperative complications were included in this study, because a significant number of patients came from other provinces and when discharged, did not come back for follow-up. The overall immediate postoperative complication rate was 2.4%, where the most common complication was bleeding that required transfusion or reoperation. According to Dindo et al.’s classification most of our postoperative complications were grade I and II. A significant number of postoperative complications were grade IIIa and IIIb, which required intervention under local or general anesthesia. Fortunately, there was no grade IV or V complications after LC in our series. Postoperative complications were similar in terms of gender, the presence of comorbidities, prior abdominal surgery, number of incisions/ports, intraoperative complications, and conversion to OC. In other studies, the postoperative complication rate has been reported from 1 to 20% [3, 4, 9–14, 16, 17]. The results of this study are comparable with those rates. As postoperative complications were significantly higher among patients classified as ASA grade III, and those with WBC count > 10,000/mm^3^ at admission in this series, it shows the importance of patient selection and preoperative management of these patients, as well as those with risk factors of intraoperative complications.

Although there was no day-case surgery in this study, the ALOS after LC was 2.23 ± 1.43 days (1–19 days), where mean hospital stay was 2.15 ± 1.3 days for LC cases and 3.67 ± 2.74 days for cases that were converted to OC. ALOS after LC was significantly higher among patients classified as ASA grade III, who had comorbidities, and those who suffered from postoperative complications. The results of this study are comparable to other studies that reported ALOS from 1.1 to 3 days [2, 4, 9, 10, 13, 16, 17]. Manning and Aziz [16] reported ALOS of 5 days in their series of 102 LCs in Cure International Hospital, Kabul, during 2006–2007 period. This study shows a significant decrease in ALOS in Cure International Hospital, Kabul after this period.

## Conclusion

While LC is the gold standard for cholelithiasis in most countries worldwide, it is less common in Afghanistan due to limited resources. However, this study shows that elective LC can be performed safely in Afghanistan with comparable outcomes in terms of complication and conversion rates and hospital stay to other countries of the region and the world. Proper case selection and careful preoperative evaluation and management can decrease further conversion, intra- and postoperative complications, and will improve the outcomes.

This is a single-center study in a major urban center, and the results are limited to this center. Since the number of cases is large, and the patients from many of Afghanistan’s provinces, it may represent a general picture of the cholelithiasis and its treatment by laparoscopic cholecystectomy in Afghanistan. Results should be interpreted with caution since follow-up after discharge was not available for some patients.

## Data Availability

Available from the corresponding author upon request.

## Abbreviations

LC: Laparoscopic cholecystectomy
OC: Open cholecystectomy
ALOS: Average length of stay
WBC: White blood cell
IRB: Institutional Review Board
ANPHI: Afghanistan National Public Health Institute
ASA: American Society of Anesthesiology
CBC: Complete blood count
HIV: Human immunodeficiency virus
LFT: Liver function test
ALT: Alanine aminotransferase
AST: Aspartate aminotransferase
FBS: Fasting blood sugar
